# Chicory Extracts and Sesquiterpene Lactones Show Potent Activity against Bacterial and Fungal Pathogens

**DOI:** 10.3390/ph14090941

**Published:** 2021-09-20

**Authors:** Suvi T. Häkkinen, Marina Soković, Liisa Nohynek, Ana Ćirić, Marija Ivanov, Dejan Stojković, Irina Tsitko, Melanie Matos, João P. Baixinho, Viktoriya Ivasiv, Naiara Fernández, Claudia Nunes dos Santos, Kirsi-Marja Oksman-Caldentey

**Affiliations:** 1 VTT Technical Research Centre of Finland Ltd., P.O. Box 1000, Tietotie 2, FI-02044 VTT Espoo, Finland; liisa.nohynek@vtt.fi (L.N.); irina.tsitko@vtt.fi (I.T.); Kirsi-Marja.Oksman@vtt.fi (K.-M.O.-C.); 2 Institute for Biological Research “Sinisa Stankovic”, National Institute of Republic of Serbia, University of Belgrade, 11000 Belgrade, Serbia; mris@ibiss.bg.ac.rs (M.S.); ana.ciric@ibiss.bg.ac.rs (A.Ć.); marija.ivanov@ibiss.bg.ac.rs (M.I.); dejanbio@ibiss.bg.ac.rs (D.S.); 3 Instituto de Tecnologia Química e Biológica António Xavier, Universidade Nova de Lisboa, Av. da República, 2780-157 Oeiras, Portugal; melanie.matos@ibet.pt; 4 iBET, Instituto de Biologia Experimental e Tecnológica, Apartado 12, 2781-901 Oeiras, Portugal; joao.baixinho@ibet.pt (J.P.B.); viktorya.ivasiv@ibet.pt (V.I.); naiara.fernandez@ibet.pt (N.F.); claudia.nunes.santos@nms.unl.pt (C.N.d.S.); 5 CEDOC, Chronic Diseases Research Centre, NOVA Medical School, Universidade NOVA de Lisboa, Campo dos Mártires da Pátria, 130, 1169-056 Lisboa, Portugal

**Keywords:** chicory, bioactivity, antimicrobial, antifungal, biofilm, cytotoxicity

## Abstract

Chicory (*Cichorium intybus* L.) is an important industrial crop cultivated mainly to extract the dietary fiber inulin. However, chicory also contains bioactive compounds such as sesquiterpene lactones and certain polyphenols, which are currently discarded as waste. Plants are an important source of active pharmaceutical ingredients, including novel antimicrobials that are urgently needed due to the global spread of drug-resistant bacteria and fungi. Here, we tested different extracts of chicory for a range of bioactivities, including antimicrobial, antifungal and cytotoxicity assays. Antibacterial and antifungal activities were generally more potent in ethyl acetate extracts compared to water extracts, whereas supercritical fluid extracts showed the broadest range of bioactivities in our assays. Remarkably, the chicory supercritical fluid extract and a purified fraction thereof inhibited both methicillin-resistant *Staphylococcus aureus* (MRSA) and ampicillin-resistant *Pseudomonas aeruginosa* IBRS P001. Chicory extracts also showed higher antibiofilm activity against the yeast *Candida albicans* than standard sesquiterpene lactone compounds. The cytotoxicity of the extracts was generally low. Our results may thus lead to the development of novel antibacterial and antifungal preparations that are both effective and safe for human use.

## 1. Introduction

Chicory (*Cichorium intybus* L. var *sativum*) is a leafy biennial or perennial plant with a strong fleshy taproot that can grow up to 75 cm in length. It belongs to the family Asteraceae, which comprises ~23,000 species that grow in temperate climate zones around the world. Chicory is an important source of the dietary fiber and industrial feedstock inulin [1,2]. It is cultivated commercially for this purpose, especially in western Europe, with an average inulin content of 17% by weight of fresh root biomass [3].

Similar to many asters, chicory also has a long history as a medicinal plant [4], which can largely be attributed to the accumulation of sesquiterpene lactones (STLs) with a broad range of health-promoting properties, including antimicrobial, anti-inflammatory and anti-cancer activities [5,6,7]. The major STLs in chicory are derived from germacrene A. The most abundant are lactucin, lactucopicrin, 8-deoxylactucin and their oxalate and glucoside derivatives [8,9]. Lactucin and lactucopicrin are responsible for the antimalarial properties of chicory. Parthenolide, another germacranolide lactone with antiparasitic activity, is produced by feverfew (*Tanacetum parthenium* (L.) Sch.Bip.) and has anti-cancer and migraine prophylaxis properties [10]. Previous work confirmed the suitability of using supercritical fluid extraction (SFE) to obtain STL from chicory roots [11].

The production of antimicrobial compounds by plants such as chicory offers a potential solution to the growing threat of antibiotic-resistant pathogens. Although the global antibiotics market is expected to reach a value of USD 62.06 billion by 2025, few compounds with novel mechanisms of action are in the development pipeline [12]. Antibiotics are used indiscriminately in vast quantities in medicine and agriculture, resulting in the emergence of pathogen strains resistant to all known antibiotics used in the clinic, and the increasing prevalence of multidrug-resistant strains of *Staphylococcus aureus*, *Pseudomonas aeruginosa*, and *Enterococcus faecium* [13]. The cost of treatment for infections caused by antibiotic-resistant bacteria increases by up to USD 40,000 per patient compared to susceptible strains [14]. The health burden of infections caused by antibiotic-resistant bacteria in the European Union is comparable to that of influenza, tuberculosis and HIV/AIDS combined, with annual costs of EUR 1.1 billion [15]. There is also a growing threat of untreatable infections. For example, vancomycin is often used as an antibiotic of last resort and is recommended to treat methicillin-resistant *Staphylococcus aureus* (MRSA) infections, but vancomycin-resistant strains have emerged over the last 20 years [16]. Novel antimicrobial compounds from plants could also help to address the drawbacks of current antifungal drugs. For example, the broad-spectrum drug amphotericin B has potent antifungal activity but causes significant nephrotoxicity-related side effects, and other synthetic antifungal agents affect the endocrine, nervous, reproductive and immune systems [17,18].

Plants synthesize many compounds with antimicrobial activity but only a small number ultimately complete clinical development and reach the market [19]. One reason for this is that pure ingredients tend to be less potent than extracts containing mixtures of antimicrobial compounds. Accordingly, the detection of antimicrobial activity in plant extracts does not always indicate the presence of a highly potent single ingredient but is more likely to reflect the synergistic activity of multiple ingredients acting on different metabolic and physiological targets in the pathogen [20]. Given that it is easier for microbes to evolve resistance to a single compound than multiple compounds with diverse targets, the development of antimicrobial extracts may offer a solution to the challenge of multidrug-resistant pathogens, and the use of well-characterized crop species increases the likelihood that such extracts will be safe for human use. Accordingly, we compared the bioactivities of different chicory extracts and pure compounds to determine the potential of this plant as a source of antimicrobial ingredients. The exploitation of chicory in this manner would also be economically advantageous by allowing the utilization of an industrial waste stream.

## 2. Results

### 2.1. Composition of the Extracts

Industrial chicory and witloof (Belgian endive) extracts containing STLs were obtained by solvent extraction and SFE. The major STLs identified in each extract are shown in Table 1. All extracts contained mixtures of 11β,13-dihydrolactucin, lactucin, 8-deoxylactucin, 11β,13-dihydro-8-deoxylactucin, 11β,13-dihydrolactucopicrin and lactucopicrin at different ratios (Supplementary Figure S1). The supercritical fluid extract fraction purified by flash column chromatography (Ci_SFE_pur_) contained a mixture of 8-deoxylactucin and 11β,13-dihydro-8-deoxylactucin. Antimicrobial activity was tested using a diverse panel of assays, focusing on typical human pathogens that are abundant on the skin and in the gut to evaluate the potential of the compounds and extracts for topical and oral application.

### 2.2. Antibacterial Activity

Chicory extracts and pure compounds generally showed higher activity against Gram-positive *Staphylococcus aureus* VTT E-70045 than Gram-negative *Escherichia coli* VTT E-94564^T^ or *Pseudomonas aeruginosa* VTT E-96728 (Table 2). The Ci_SFE extract showed strong antibacterial activity, whereas the witloof and chicory ethyl acetate extracts (Wit_EtOAc and Ci_EtOAc) showed moderate activity, and the witloof water extract (Wit_H_2_O) showed only weak activity. The pure compounds lactucopicrin, parthenolide and 11β-13-dihydrolactucopicrin moderately inhibited the growth of *S. aureus*, whereas lactucin was a weak inhibitor (Table 2). All the standard compounds showed weak antimicrobial activity against *E. coli* and *P. aeruginosa.*

Witloof was used to assess the most suitable extraction solvent for optimized bioactivity. The strongest antimicrobial activity against *S. aureus* VTT E-70045 was achieved using ethyl acetate (Wit_EtOAc) and SFE (Ci_SFE), resulting in moderate and strong growth inhibition, respectively, at concentrations of 1 mg/mL (Supplementary Figure S2). The final bacterial colony forming unit (CFU) counts in the *S. aureus* cultures after incubation for 48 h differed from control cultures by 1 (Wit_EtOAc) and 1.5 (Ci_SFE) log units, indicating > 90% growth inhibition. Two chicory extracts were prepared from fresh industrial chicory root using ethyl acetate (Ci_EtOAc) or water (Ci_H_2_O) as solvents. As observed for the pure compounds, Ci_EtOAc showed weak activity against *E. coli* and *P. aeruginosa* (growth inhibition < 5%) at a concentration 0.05 mg/mL in dimethylsulfoxide (DMSO) but a stronger effect against *S. aureus* (growth inhibition = 20%). Ci_H_2_O showed weak activity against *S. aureus*. The water extracts contained lactucin, 11β,13-dihydrolactucin, 8-deoxylactucin and 11β,13-dihydro-8-deoxylactucin, whereas the ethyl acetate extracts mainly contained 11β,13-dihydrolactucin, 8-deoxylactucin, 11β,13-dihydro-8-deoxylactucin and 11β,13-dihydrolactucopicrin (Supplementary Figure S1). Neither of the extracts showed antimicrobial activity against *S. aureus* MRSA. The results with witloof and industrial chicory extracts indicated that higher antimicrobial activity can be achieved by extraction with ethyl acetate than water, but that SFE was the most promising method.

The same extracts and standard compounds were tested using the microdilution method. In agreement with our earlier results, Ci_EtOAc showed antimicrobial activity against all the test pathogens except the resistant *P. aeruginosa* strain IBRS P001, whereas Ci_H_2_O showed no antimicrobial activity at the concentrations we tested (Table 3). The standards (parthenolide and lactucopicrin) showed slightly better antimicrobial activity than Ci_EtOAc, with minimal inhibitory concentrations (MICs) in the range 0.08–0.50 mg/mL. Only costunolide and 11β-13-dihydrolactucin were active against *P. aeruginosa* IBRS P001 (MIC = 0.5 mg/mL) and none of the standards inhibited *E. coli* (Table 3). However, weak activity was observed in the original assay (Table 1), probably reflecting the use of different bacterial strains (*E. coli* ATCC 25922 and VTT E-94564T, respectively). Lactucin showed no activity against any of the tested bacteria. Commercial antibiotics were used as controls and showed better activity than the extracts and standards.

**Figure 1:**
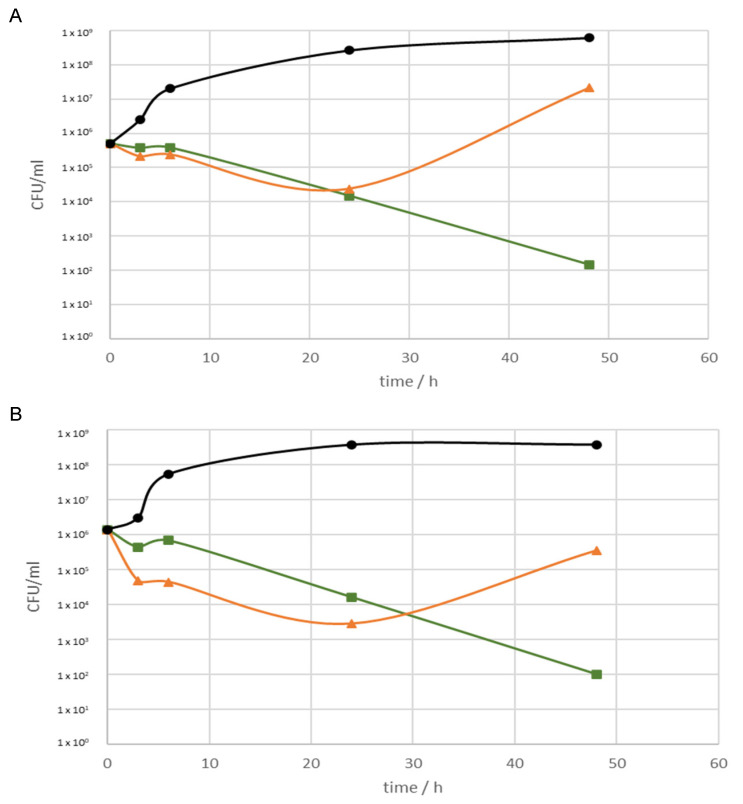
Growth inhibition by Ci_SFE_opt_ and Ci_SFE_pur_ at a concentration of 5 mg/mL against (**A**) *S. aureus*, and (**B**) *S. aureus* MRSA. Green, Ci_SFE_pur_; orange, Ci_SFE_opt_, black, microbial control.

Ci_SFE_opt_ and particularly Ci_SFE_pur_ showed potent antimicrobial activity against *S. aureus* and *S. aureus* MRSA (Figure 1). Ci_SFE_pur_ showed a stronger inhibitory effect over time, whereas Ci_SFE_opt_ was most active during the first 24 h followed by the moderate recovery of bacterial growth. These extracts had no effect against *P. aeruginosa* (data not shown). Microdilution assays revealed promising antibacterial properties for Ci_SFE_pur_ and Ci_SFE_opt_, with MICs of 0.06–0.25 and 0.5–2.1 mg/mL, respectively (Table 4). Ci_SFE_pur_ appeared more potent than Ci_SFE_opt_. The most sensitive bacterial species was *P. aeruginosa* IBRS P001 (MIC Ci_SFE_pur_ = 0.06 mg/mL; MIC Ci_SFE_opt_ = 0.50 mg/mL). The activity of the extracts against the antibiotic-resistant *S. aureus* MRSA and *P. aeruginosa* IBRS P001 strains is particularly noteworthy given the urgent need for new drugs against antibiotic-resistant pathogens.

**Figure 2:**
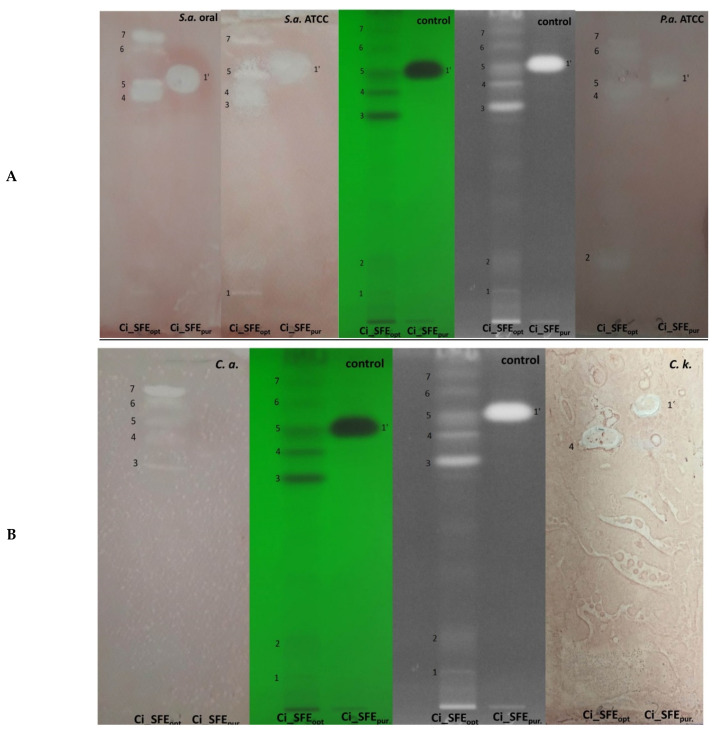
Bioautographic assay of Ci_SFE_opt_ and Ci_SFE_pur_ developed in 85:15 (*v*/*v*) dichloromethane:methanol as the solvent. (**A**) Antibacterial activity against *Staphylococcus aureus* (oral isolate), *S. aureus* ATCC (left); and *Pseudomonas aeruginosa* (right). (**B**) Antifungal activity against *Candida albicans* (left) and *C. krusei* (right). Spots on different Rf values: 1. Rf = 0.18 (activity only against *S. aureus* ATCC); 2. Rf = 0.20 (activity only against *P. aeruginosa*); 3. Rf = 0.65 (activity against *S. aureus* ATCC and *C. albicans*); 4. Rf = 0.71 (activity against all tested bacteria and fungi); 5. Rf = 0.76 (activity against all tested bacteria and fungi, except *C. krusei*); 6. Rf = 0.82 (activity against all tested microorganisms, except *P. aeruginosa* and *C. krusei*); 7. Rf = 0.88 (activity against all tested bacteria and fungi, except *C. krusei*).

Next, we used thin layer chromatography (TLC) bioautography to screen our extracts and compounds for their biological activity. Ci_SFE_opt_ was active against all tested microorganisms with R_f_ values ranging from 0.18 to 0.88 (Figure 2A,B). The purified SFE fraction (Ci_SFE_pur_, R_f_ = 0.76) was active against the *S. aureus* oral isolate, *S. aureus* ATCC, *P. aeruginosa* and also the fungus *Candida krusei*.

### 2.3. Antifungal Activity

The development of antifungal drugs is challenging because both the pathogen and host are eukaryotic, limiting the number of suitable molecular targets. Drug-resistant fungal strains have also been reported, including *Candida* species resistant to the common antifungal fluconazole [21]. We found that all our compounds and extracts inhibited the growth of the four *Candida* species we tested, as well as two strains of *C. albicans* (MIC = 0.03–1.00 mg/mL) (Table 5). The most potent extracts were Ci_SFE_opt_ and Ci_SFE_pur_, which inhibited all five fungi in the test panel and achieved a MIC of 0.03 mg/mL against *C. auris*, which was lower than the MIC of any of the pure compounds. None of the tested standards showed activity against micromycetes (Table 5). Ci_EtOAc showed antifungal activity against *Aspergillus versicolor*, *A. ochraceus*, *A. niger*, *Trichoderma viride* and *Penicillium ochrochloron*, whereas Ci_H_2_O was moderately active solely against *C. albicans* (Table 5). Ci_SFE_opt,_ and Ci_SFE_pur_ showed particularly potent activity against *A. fumigatus, A. versicolor* and *A. ochraceus*, with Ci_SFE_pur_ achieving MICs lower than the commercial antifungal agent ketoconazole (Table 5).

### 2.4. Antibiofilm Activity

**Figure 3:**
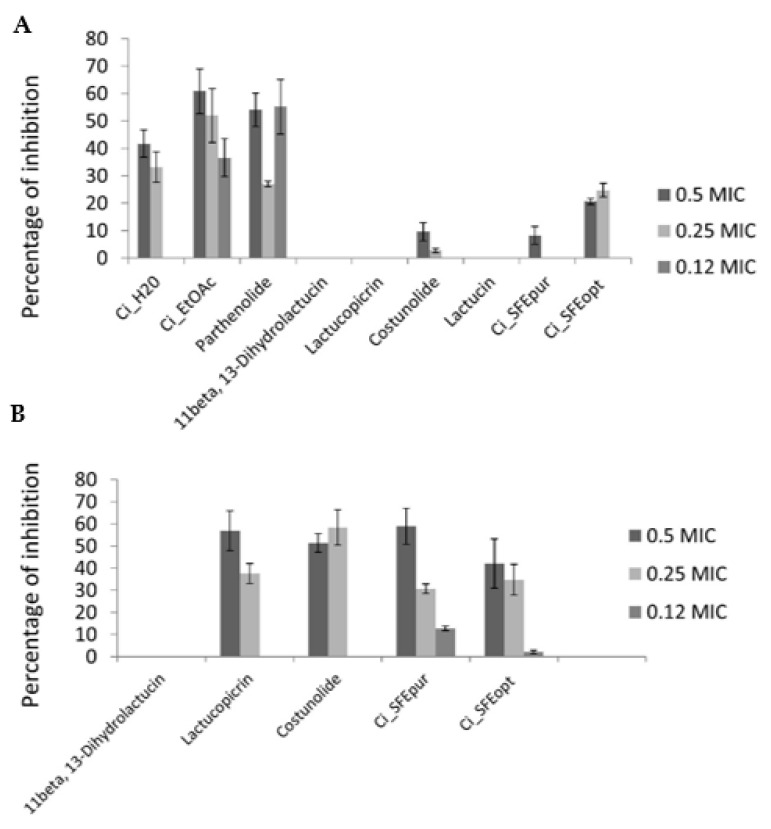
Percentage inhibition of biofilm formation in (**A**) *C. albicans* 475/15 and (**B**) *P. aeruginosa* IBRS P001 after treatment with chicory extracts and pure compounds. Results are presented as a mean (± stdev) of triplicates.

Chicory extracts demonstrated higher antibiofilm activity against *C. albicans* than standard sesquiterpene lactone compounds. However, Ci_EtOAc and Ci_H_2_O achieved higher antibiofilm activities than Ci_SFE_opt_ and Ci_SFE_pur_ (Table 5), despite the more potent antifungal activities of the supercritical fluid extracts (Figure 3A). Ci_EtOAc showed the most promising antibiofilm activity against *C. albicans*, slightly higher than the activity of parthenolide. The antibiofilm activities of the chicory extracts were relatively high compared to the antifungal agent ketoconazole, which achieved 73% inhibition at 0.5 MIC (Figure 3A). Compounds 11β-13-dihydrolactucin, lactucopicrin, lactucin and costunolide did not show antibiofilm activity at the tested subinhibitory concentrations, whereas the activities of Ci_SFE_pur_ and Ci_SFE_opt_ were slightly higher but not significantly.

Given the promising antibacterial activity of the supercritical fluid extracts against *P. aeruginosa* IBRS P001 (Table 3), the same extracts were also tested for their antibiofilm activity against this bacterial pathogen. Remarkably, Ci_SFE_pur_ achieved almost 60% inhibition at 0.5 MIC (Figure 3B). Lactucopicrin and costunolide also reduced the formation of *P. aeruginosa* IBRS P001 biofilms by ~50% at 0.5 MIC, whereas 11β-13-dihydrolactucin showed no antibiofilm activity (Figure 3B). Based on the results of crystal violet assays and the ability of CI_SFE_opt_ and Ci_SFE_pur_ to reduce *P. aeruginosa* biofilm biomass, we selected these extracts for further antibiofilm assessment.

**Figure 4:**
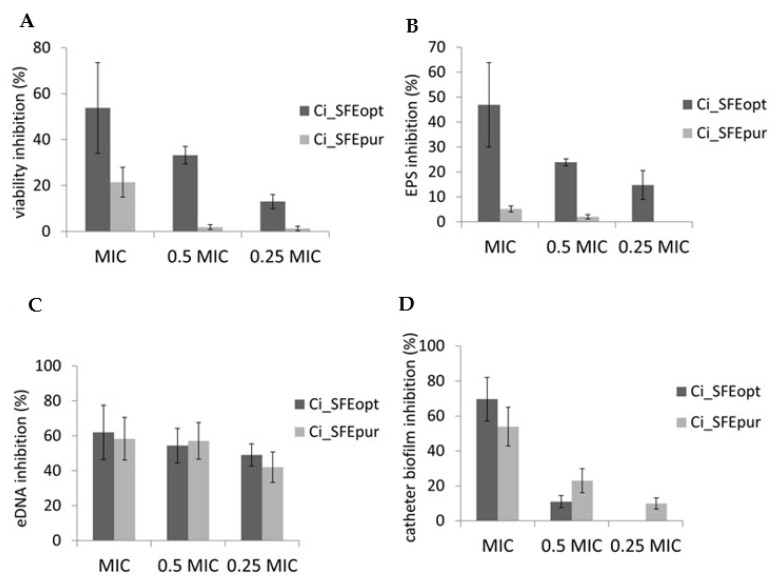
Modes of antibiofilm activity against *P. aeruginosa* IBRS P001 expressed as percentage inhibition compared to the untreated control. (**A**) Inhibition of biofilm cell viability determined by MTT assay. (**B**) Inhibition of biofilm exopolysaccharide content. (**C**) Inhibition of biofilm eDNA. (**D**) Inhibition of biofilm CFU formed on urinary catheters. Results are expressed as mean (±stdev) of triplicates.

MTT assays revealed that biofilm cell viability was significantly reduced (> 50%) following Ci_SFE_opt_ treatment, whereas Ci_SFE_pur_ had a less profound effect (Figure 4A). Biofilms are composed of microbial cells embedded in an extracellular matrix with exopolysaccharides (EPS) and extracellular DNA (eDNA) as essential components; thus, we investigated the effect of Ci_SFE_opt_ and Ci_SFE_pur_ on these factors. Ci_SFE_opt_ (but not Ci_SFE_pur_) was able to reduce the quantity of EPS in the biofilm (Figure 4B), whereas both extracts reduced the quantity of eDNA (Figure 4C). Given that *P. aeruginosa* biofilms are often present in catheter-associated infections, we determined whether the incubation of catheters in MIC and sub-MIC amounts of Ci_SFE_opt_ and Ci_SFE_pur_ would reduce the ability of *P. aeruginosa* to establish biofilms on the catheter surface. Both Ci_SFE_opt_ and Ci_SFE_pur_ reduced the CFU count when applied at the MIC, with Ci_SFE_opt_ showing the stronger inhibitory potential (Figure 4D).

The extracts Ci_SFE_opt_ and Ci_SFE_pur_ differ in the active compound spectra, which may result in the more profound antibiofilm effects in the cases of Figure 4A,B, i.e., putative synergistic action of higher number of active compounds may play a role, whereas in the case of biofilm eDNA (Figure 4C) inhibition by only purified fraction compounds are sufficient to perform the action. Accordingly, antibiofilm activity is not strongly correlated with eDNA. eDNA inhibition is one of the many mechanisms to reduce integrity of the biofilm structure and is one of the causes of biofilm resistance to antimicrobials. It is not surprising that both extracts exhibited the similar action on eDNA inhibition, although Ci_SFE_opt_ had more pronounced antibiofilm activity.

### 2.5. Inhibition of Pyocyanin Production by Pseudomonas aeruginosa IBRS P001

Pyocyanin is a *Pseudomonas* toxin that confers a competitive advantage by killing other microbes, but it also kills mammalian cells. The inhibition of pyocyanin synthesis helps to eradicate *P. aeruginosa* and can also prevent infection. The sesquiterpene lactones were tested at 0.5 MIC for their ability to inhibit pyocyanin production by *P. aeruginosa* (Table 6). Based on the concentration of pyocyanin (mg/mL) in treated bacterial cultures, lactucin demonstrated the strongest effect (~70% inhibition) and 11β-13-dihydrolactucin the weakest (~36% inhibition). The strong effect of these compounds on pyocyanin production exists at sub-MIC levels.

### 2.6. Toxicity

Acute toxicity was assessed using a photobacterial bioluminescence assay (*Aliivibrio fischeri*), which is a highly sensitive method for the detection of atmospheric pollutants, heavy metals and industrial effluents [22]. The chicory samples assessed in this study were in most cases not harmful (Table 7). Wit_EtOAc formed precipitates on the walls of the reaction tube; thus, the sample was sonicated after dilution in 2% NaCl and the toxicity test was repeated. Sonication reduced the EC_50_ value slightly, indicating that the precipitates may have contained moderately toxic components. Among the three supercritical fluid extracts, Ci_SFE and Ci_SFE_opt_ were the most toxic toward *A. fischeri*, probably reflecting the synergetic effect of multiple compounds.

We next assessed the antiproliferative effect of Ci_SFE_opt_ and Ci_SFE_pur_ in the immortalized human keratinocyte line HaCaT, which is widely used as an in vitro model for initial compound screening before progressing to skin irritation tests. Ci_SFE_opt_ showed weak cytotoxicity (IC_50_ = 303.42 µg/mL) whereas Ci_SFE_pur_ was highly cytotoxic (IC_50_ = 16.76 µg/mL), suggesting that Ci_SFE_opt_ is safe to use with caution whereas Ci_SFE_pur_ is potentially a potent skin irritant (Table 8). These results should be confirmed in vivo. Potassium dichromate (K_2_Cr_2_O_7_) was used as positive control and showed the strongest effect in the assay.

**Figure 5:**
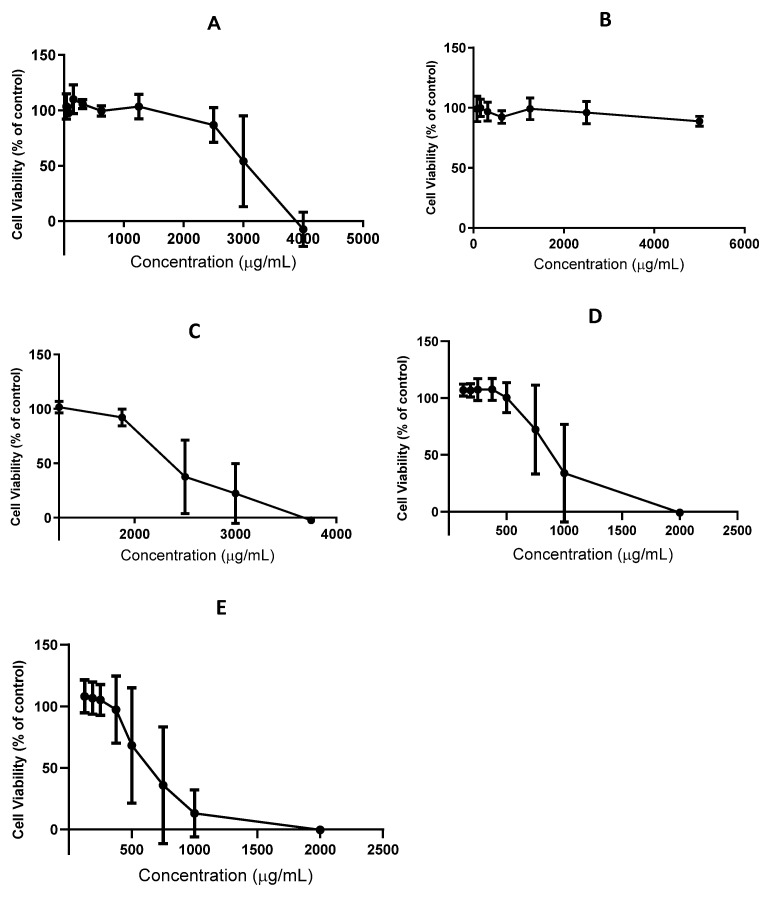
Cytotoxic profile of chicory extracts in Caco-2 cells after incubation for 4 h. (**A**) Wit_EtOAc. (**B**) Wit_H_2_O. (**C**) Ci_EtOAc. (**D**) Ci_SFE_opt_. (**E**) Ci_SFE_pur_. Results are presented as mean (±stdev) of three independent experiments.

We also tested the effect of witloof and chicory extracts in Caco-2 cells, which are the preferred in vitro model of the intestinal epithelial barrier. In initial experiments, the cells were exposed for 4 h (the commonly accepted period for gastrointestinal traffic) and subsequently for 24 h, but the toxicity of the extracts was not enhanced by the longer incubation. The witloof ethyl acetate extract (Wit_EtOAc) demonstrated cytotoxicity at concentrations exceeding 2500 μg/mL (Figure 5A), whereas the aqueous extract (Wit_H_2_O) showed no significant cytotoxicity until the concentration reached 5000 μg/mL (Figure 5B). This is probably because water extracts the more polar compounds from witloof, including sugars and inulin, which are not cytotoxic at the concentrations we tested. The Ci_EtOAc was cytotoxic at concentrations > 1875 μg/mL, whereas Ci_SFE_opt_ already showed cytotoxicity at 750 μg/mL (Figure 5C,D). The higher cytotoxicity of the supercritical fluid extract reflected the higher selectivity of the SFE process for STL extraction, given that the mass percentage of these compounds is higher in this extract compared to Ci_EtOAc. Ci_SFE_pur_ is highly enriched for 8-deoxylactucin and 11β,13-dihydro-8-deoxylactucin, and this was more cytotoxic than Ci_SFE_opt_, affecting cell viability starting at a concentration of 375 μg/mL (Figure 5E). This higher toxicity is likely to be associated with the purification of the most bioactive compounds from Ci_SFE_opt_, which overcomes the dilution effect often observed in more complex mixtures.

## 3. Discussion

Natural compounds remain an important source of new drugs. During the period 1981–2019, up to 67% of all approved small molecules were natural products or contained an active pharmacophore from a natural product [23]. Most current antibiotics are microbial metabolites, but plant-derived compounds show promising activities and the number of scientific publications on this topic increases continually [24,25]. Among the 459 antibiotic compounds identified in plants, 50.8% are phenolic derivatives and 26.6% are terpenoids [26]. In our study, we detected several interesting bioactivities in extracts and purified compounds from chicory, an industrial crop that is not presently used for medicinal purposes. Importantly, the bioactive fraction can be obtained from an industrial waste stream. We prepared witloof and chicory extracts rich in STLs and tested them for different bioactivities. Conventional solid–liquid extraction with water was generally less selective for STLs than ethyl acetate, as reflected by the weaker bioactivity of the aqueous extracts. But SFE was more selective than ethyl acetate, achieving the highest overall STL yields. We found that the further optimization of SFE yielded chicory extracts with strong antibacterial and antifungal activity, and others have likewise reported chicory extracts with potent anti-inflammatory activity using a similar SFE method [11]. The mixture of natural compounds found in plant extracts often demonstrates synergistic effects, as we observed for our extracts. Whereas pure STLs demonstrated modest antifungal activity, the chicory extracts containing mixtures of STLs showed strong antifungal effects. Remarkably, the chicory supercritical fluid extract and a purified fraction consisting of 8-deoxylactucin and 11β,13-dihydro-8-deoxylactucin inhibited the growth of both antibiotic-resistant bacteria tested in this study (*S. aureus* MRSA and *P. aeruginosa* IBRS P001). Antibiotic resistance is a major global health challenge that causes an immense burden on health care systems [13]. During the past 20 years, only two new antibiotic classes (lipopeptides and oxazolidinones) have been approved that are suitable for the treatment of antibiotic-resistant bacteria, and both target Gram-positive species [27]. Among the 44 intravenous antibiotics in the clinical development pipeline, only 15 show activity against Gram-negative species such as *P. aeruginosa* IBRS P001, and only five have progressed to phase III trials [28,29]. The *S. aureus* MRSA strain used in this study is ranked as 13th on the WHO priority list of the 25 most severe pathogenic bacteria [29]. 

All current clinical antibiotics are either bactericidal or bacteriostatic, and growth inhibition is achieved by interfering with essential cellular processes [30]. The resulting selective pressure leads to the emergence of resistant strains, which rapidly spread in the population. This can be addressed by focusing on the development of drugs that limit selective pressure by targeting nonessential cellular processes, such as bacterial virulence, quorum sensing, or the ability to form biofilms [26,31,32]. The formation of biofilms during chronic infections is also one of several mechanisms used by pathogenic bacteria to withstand antibiotics. We found that chicory extracts, especially those obtained by SFE, were able to inhibit biofilm formation by *P. aeruginosa* IBRS P001. The extracts reduced biofilm cell viability as well as the accumulation of EPS and eDNA in the extracellular matrix. Further investigation revealed that chicory supercritical fluid extracts are promising inhibitors of catheter-related biofilms. 

The development of new antifungal agents safe for human use is challenging because fungi and mammals are both eukaryotes and thus share many drug targets. The major life-threatening fungal infections in humans are caused by species of *Candida*, *Aspergillus* and *Cryptococcus* [33]. Only three types of antifungal drugs are currently available, and this limited arsenal is threatened by the emergence of multidrug-resistant pathogens such as *C. auris*, first reported in 2009 in Japan [34]. *C. auris* spreads rapidly in critically ill patients to become a dominant opportunistic pathogen; thus, there is an urgent need for new antifungal agents [35]. The strong antifungal activity of the chicory supercritical fluid extract against *C. auris* is therefore an important step forward, particularly given the ability of the purified extract to inhibit biofilm formation by *C. albicans* more effectively than standard STLs. 

The therapeutic index of a drug is a measure of its relative safety and efficacy, with a high score indicating the ideal situation in which a drug simultaneously shows high activity against its target but low toxicity. In an initial acute toxicity screen, only the crude supercritical fluid extract showed cytotoxicity, which declined when the SFE method was optimized and with the fractionated extracts. A higher mass percentage of STL compounds (as found in the supercritical fluid extracts) correlated with higher cytotoxicity in HaCaT and Caco-2 cells, highlighting the dose-dependent bioactivity and toxicity of these compounds.

Natural compounds offer a large and largely untapped resource of novel drug candidates [36]. Our results demonstrate that chicory is a source of valuable bioactive compounds with antimicrobial properties, and the isolation of chicory extracts and compounds from industrial waste streams has the potential to create added value that can be exploited to benefit human health. The same principles can be applied to many other underutilized plants as well as plant-derived biomass generated as side streams or waste from industrial processes. 

## 4. Materials and Methods

### 4.1. Sample Extraction

We tested seven samples consisting of witloof or industrial chicory extracted with different solvents. Freeze-dried witloof and fresh industrial chicory roots were extracted with ethyl acetate (Wit_EtOAc, Ci_EtOAc) or water (Wit_H_2_O, Ci_H_2_O) at a raw material to solvent ratio of 1:10 (*w*/*v*). Extraction was performed for 1 h at 60 °C, with constant agitation at 900 rpm in an RW20.n mechanical stirrer (IKA, Staufen, Germany). Each extract was passed through FILTER-LAB 125-mm qualitative filter paper (Scharlab, Barcelona, Spain) before remaining particulates were removed by centrifugation in a model 5810 R benchtop device (Eppendorf, Hamburg, Germany). The extracts were then dried in a rotary evaporator under reduced pressure at 40 °C. SFE was applied to freeze-dried industrial chicory (Ci_SFE) at a flow rate of 10 g/min CO_2_ with 10% ethanol as the co-solvent (300 bar, 50 °C, 120 min). After SFE process optimization for freeze-dried industrial chicory (Ci_SFE_opt_), extracts were prepared at a flow rate of 15 g/min CO_2_ with 10% ethanol as the co-solvent (350 bar, 40 °C, 120 min). The Ci_SFE_opt_ extract and Ci_SFE_pur_ (purified fraction) were prepared as previously described [11]. 

### 4.2. Identification of STLs

STLs were identified by HPLC on a Dionex Ultimate 3000 device equipped with a quaternary pump, solvent degasser, autosampler, and column oven, coupled to a Dionex DAD-3000 photodiode array detector (all from Thermo Fisher Scientific, Waltham, MA, USA). Samples were fractionated on a LiCrospher 100 RP-18, 250 mm × 4 mm (5 µm) reversed-phase column (Merck, Darmstadt, Germany) at 35 °C, and were eluted in a gradient of mobile phase A (14:86 (*v*/*v*) methanol/water) and mobile phase B (64:36 (*v*/*v*) methanol/water). The gradient program was 0 to 20 min, 100–58% A; 20 to 30 min, 58% A; 30 to 45 min, 58–0% A; 45 to 50 min, 0% A; 50 to 52 min, 0–100% A; 52 to 62 min, 100% A. The flow rate was 0.5 mL/min, and the injection volume was 20 µL. A photodiode array detector was used to scan for absorption in the range 210–600 nm. Data were analyzed using Chromeleon v7.2 SR4. Ethanol was the preferred solvent for sample preparation.

### 4.3. Antimicrobial Activity

Antimicrobial activity in cultures spiked with terpene compounds and extracts was tested as previously described [37] with modifications [38], or using an automated Bioscreen turbidimeter (Labsystems, Helsinki, Finland) [39]. Pure standard compounds (lactucopicrin, 11β-13-dihydrolactucopicrin, lactucin and parthenolide) and extracts were tested against *S. aureus* VTT E-70045 (ATCC 6538), *P. aeruginosa* VTT E-96728 (ATCC 9027) and *E. coli* VTT E-94564^T^ (ATCC 11775) in Mueller-Hinton broth (Sigma-Aldrich, St Louis, MO, USA). Stock solutions of the standard compounds (4 mM) were prepared in DMSO. Microbial growth was monitored in duplicate at 37 °C for 48 h. Each sample was analyzed as duplicates.

The antimicrobial activity of the samples was also tested by analyzing the type of growth inhibition (biostatic or biocidal) using a modified microdilution method [40] and bioautography on TLC plates [41]. We tested *S. aureus* (oral isolate), *S. aureus* ATCC 11632, *P. aeruginosa* ATCC 27853, *P. aeruginosa* IBRS P001, and *E. coli* 25922. We also tested Ci_SFE_opt_ and Ci_SFE_pur_ against *Proteus mirabilis* ATCC 7002, *Listeria monocytogenes* NCTC 7973, *Yersinia enterocolitica* ATCC 23715, *Klebsiella pneumoniae* ATCC 13883, *Campylobacter jejuni* ATCC 33560, and *Enterobacter cloacae* human isolate. Briefly, bacterial suspensions were adjusted with sterile saline to a concentration of 1.0 × 10^5^ CFU/mL. Extracts and compounds dissolved in 30% ethanol were added in 100 μL tryptic soy broth (TSB) to a bacterial inoculum of 1.0 × 10^4^ CFU per well. The highest test concentration was 8 mg/mL for the extracts and 1 mg/mL for the compounds. The lowest concentrations that completely inhibited bacterial growth (MICs) were determined visually under a binocular microscope and also by a colorimetric microbial viability assay based on the reduction of *p*-iodonitrotetrazolium violet (Sigma-Aldrich) compared to positive control compounds for each bacterial strain. MBCs were determined by the serial dilution of 2 μL culture samples into microtiter plates containing 100 μL of fresh medium per well, followed by incubation for 24 h. The lowest concentration with no visible growth was defined as the MBC, indicating the death of 99.5% of cells from the original inoculum. Ampicillin and streptomycin were used as positive controls, and the solvent ethanol as a negative control. All tested species are available at the Mycological Laboratory, Department of Plant Physiology, Institute for Biological Research “Siniša Stankovic”, University of Belgrade.

In the modified bioautographic method [41], we applied 10 µL of Ci_SFE_opt_ and Ci_SFE_pur_ (2 mg/mL in 30% ethanol) to a Kieselgel 60 F254 TLC plate (Merck) and fractionated the duplicate samples using an 85:15 (*v*/*v*) mixture of dichloromethane and methanol as the mobile phase. The position of the first tape was 1.50 cm from the left and 1.00 cm from the bottom. The distance between the tapes was 1.30 cm. The development time of the panel was 18 min, and the separation path was 8.5 cm in length. The absorbent layers were dried in an oven at 90 °C for 5 min to remove the solvent. One of the strips was visualized under UV light and the second strip was used for the bioautography assay. The R_f_ value of each spot was measured. The dried plates were sprayed with freshly prepared cultures (1.0 × 10^5^ CFU/mL in TSB) of *S. aureus* (oral isolate), *S. aureus* (ATCC 11632), *P. aeruginosa* (ATCC 27853), *C. albicans* (oral isolate) and *C. krusei* (oral isolate). The plates were incubated in a water–vapor chamber for 24 h at 37 °C before spraying with 3% *p*-iodonitrotetrazolium violet. After storage for 3 h, the plates were sprayed with 70% ethanol to stop bacterial and fungal growth. Microbial growth inhibition appeared as clear zones against a pink background and the R_f_ values of the spots showing inhibition were determined. 

### 4.4. Antifungal Activity

We determined the MIC and minimal fungicidal concentrations (MFC) of the compounds against *A. fumigatus* ATCC 9197, *A. niger* ATCC 6275, *A. versicolor* ATCC 11730, *P. funiculosum* ATCC 36839, *C. albicans* 475/15 (clinical isolate), *C. albicans* ATCC 10231, *C. krusei* H1/16 (clinical isolate), *C. auris* ATCC B 11903 and *C. parapsilosis* ATCC 22109 by applying the microdilution technique in 96-well microtiter plates [42] with modifications. As above, the highest test concentration was 8 mg/mL for extracts and 1 mg/mL for pure compounds. Briefly, fungal cultures were diluted in sterile saline to ~1.0 × 10^5^ CFU/per well. MIC and MFC values were determined by incubating fungal cells in Sabouraud dextrose broth (SDB, Merck) with serial dilutions of compounds at 37 °C for 24 h (yeast) and at 25 °C for 72 h (filamentous fungi). The MIC was the lowest concentration of each compound at which no visible fungal growth was observed by microscopy. After serial sub-cultivation by transferring 10 µL of each culture to microtiter plates containing 100 µL SDB/well, followed by incubation as above, MFC values were the lowest concentrations at which no visible fungal growth was observed by microscopy, indicating the death of 99.5% of the original inoculum. Fungal isolates were maintained on Sabouraud dextrose agar (Merck) at 4 °C and were subcultured monthly. Ketoconazole (Sigma-Aldrich) was used as a positive control and the solvent ethanol as a negative control.

### 4.5. Antibiofilm Activity

#### 4.5.1. Calculation of Percentage Inhibition

Antibiofilm assays were conducted as previously described [43], with modifications, using *C. albicans* 475/15 growing in yeast extract–peptone–dextrose (YPD) medium (Merck) and *P. aeruginosa* IBRS P001 [44] growing in TSB + 2% glucose (Torlak Institute of Immunology and Virology, Belgrade, Serbia)*. C. albicans* and *P. aeruginosa* were incubated in 96-well microtiter plates with adhesive bases (Sarstedt, Nümbrecht, Germany) at 37 °C with sub-MIC concentrations of each compound. After 24 h, each well was washed twice with sterile phosphate buffered saline (PBS, pH 7.4) and the adherent cells were fixed in methanol. The plates were then air dried and the cells were stained with 0.1% crystal violet (bioMerieux, Marcy-l’Étoile, France) for 30 min. The wells were washed with PBS to remove excess stain and air dried before adding 100 µL 96% ethanol (Zorka Pharma, Šabac, Serbia) to solubilize the stain. Absorbance at 620 nm was measured on a Multiskan FC microplate photometer (Thermo Fisher Scientific) and the percentage inhibition of biofilm formation was calculated using the following equation:

%Inhibition = [(A620_control_ − A620_sample_)/A620_control_] × 100.

#### 4.5.2. Biofilm Cell Viability MTT Assay

The effect of Ci_SFE_opt_ and Ci_SFE_pur_ on *P. aeruginosa* IBRS P001 biofilm formation was determined by assessing the metabolic activity of live cells using an MTT assay [45]. Biofilms were allowed to develop for 24 h at 37 °C in the presence of Ci_SFE_opt_ and Ci_SFE_pur_ (0.25–1.0 MIC). The supernatants were then removed and the biofilms in the microtiter plates were washed with PBS before adding 200 μL MTT reagent (0.5 mg/mL) and incubating at 37 °C in the dark for 2 h. The resulting purple dye was dissolved in 200 μL DMSO and the absorbance was measured at 570 nm in a microtiter plate reader. The percentage inhibition of biofilm cell viability was calculated using the following equation:

%Inhibition = [(A570_control_ − A570_sample_)/A570_control_] × 100.

#### 4.5.3. Congo Red EPS-Binding Assay

EPS production by *P. aeruginosa* IBRS P001 biofilm was measured as previously described ^44^, with modifications. Biofilms were formed in the presence of Ci_SFE_opt_ and Ci_SFE_pur_ (0.25–1 MIC) for 24 h at 37 °C. The planktonic cells were then discarded, and the adherent cells were washed with PBS and stained with 1% (*w*/*v*) Congo red in the dark for 30 min. Excess dye was removed and the bound dye was solubilized with 200 μL DMSO. Absorbance at 490 nm was measured in a microtiter plate reader and the percentage inhibition of EPS production was calculated using the following equation:

%Inhibition = [(A490_control_ − A490_sample_)/A490_control_] × 100

#### 4.5.4. Quantification of eDNA

The quantity of eDNA was determined as previously described [46], with modifications. *P. aeruginosa* was grown in 96-well plates containing 200 µL TSB + 2% glucose per well, with and without Ci_SFE_opt_ or Ci_SFE_pur_ (0.25–1.0 MIC). After incubation at 37 °C for 24 h, the planktonic cells were removed, and the wells were washed with PBS. We then added 150 µL TE buffer to the wells and mixed vigorously by pipetting. The solution was transferred to 1.5-mL tubes and centrifuged at 10,000× *g* for 10 min. The supernatant was removed, and the pellet resuspended in 100 μL TE buffer by vortexing before centrifugation at 10,000× *g* for 15 min. The amount of eDNA was quantified by measuring the absorbance at 260 nm, with Milli-Q water as the blank. We calculated percentage depletion of eDNA relative to an untreated control.

#### 4.5.5. Catheter Model of Biofilm Formation

Sterile silicon 16-mm Romed catheters (Van Oostveen Medical, Wilnis, The Netherlands) were cut into 1 cm lengths and placed in 24-well plates. We then added *P. aeruginosa* IBRS P001 in TSB + 2% glucose and the test compounds (0.25–1.0 MIC). After incubation at 37 °C for 24 h, the catheters were washed with PBS and transferred to 1.5 mL Eppendorf tubes before adding 1 mL PBS and vortexing vigorously. The samples were diluted, seeded onto plate-count agar (HiMedia Laboratories, Mumbai, India) and incubated at 37 °C for 24 h. We then determined the CFU count and determined the percentage inhibition relative to the untreated control.

### 4.6. Inhibition of Pseudomonas aeruginosa IBRS P001 Pyocyanin Production

Overnight cultures of *P. aeruginosa* (10^9^ CFU) in 1 mL lysogeny broth (LB) were incubated at 37 °C for 48 h in the presence of each compound (0.5 MIC). The treated culture was centrifuged, and the supernatant was extracted with chloroform and mixed with 0.2 M HCl. We measured the absorbance of the organic layer at 520 nm using a UV1601 spectrophotometer (Shimadzu, Kyoto, Japan). The inhibition of pyocyanin production compared to untreated controls was calculated using the following equation:

%Inhibition = [(A520_control_ − A520_sample_)/A520_control_] × 100.

The standard pyocyanin compound was serially diluted and the absorbance was measured as described above. Concentrations of pyocyanin in the control and treated cultures were presented in mg/mL using a calibration curve for standard compounds.

### 4.7. Toxicity

#### 4.7.1. Photobacterial Acute Toxicity Assay

Acute toxicity tests followed the ISO 21338 standard method adapted to the 96-well plate format. We used the BioTox kit (Aboatox, Turku, Finland) with freeze-dried *A. fischeri* according to the supplier’s instructions, with minor modifications. Before each test, the freeze-dried bacteria were rehydrated and stabilized at 4 °C for 1 h, followed by 1 h at 10 °C and final stabilization at 20 °C for 10 min. We used 96-well flat-bottom polystyrene microtiter plates at ambient temperature (20–21 °C). Bioluminescence was measured in a Fluoroscan Ascent FL microplate automated luminometer (ThermoLabsystems, Helsinki, Finland) equipped with an automatic dispenser and mixer. Sample stocks were prepared in DMSO, unless otherwise stated. We pipetted 140 μL of sample into each well, followed by the automatic addition of 140 μL bacterial suspension. Luminescence was recorded continuously for 6 s, the maximum was noted, and the results were normalized to the peak value. The measurements were repeated after 15 and 30 min. Toxicity was expressed as the effective concentration causing 50% bioluminescence inhibition (EC_50_) as recommended by ISO 21338. Unless otherwise stated, duplicate toxicity assays were prepared for eight serial two-fold dilutions of each sample. Each run included 3,5-dichlorophenol as a positive control.

#### 4.7.2. HaCaT Cell Antiproliferative Activity Assay

The antiproliferative effect of Ci_SFE_opt_ and Ci_SFE_pur_ was determined in HaCaT cells grown in high-glucose Dulbecco’s Modified Eagle’s Medium (DMEM) supplemented with 10% fetal bovine serum (FBS), 2 mM l-glutamine and 1% penicillin and streptomycin (Invitrogen, Thermo Fisher Scientific, Waltham, MA, USA) at 37 °C in a 5% CO_2_ atmosphere. We seeded 1 × 10^4^ cells/well in a 96-well plate 24 h before treatment. The medium was removed and replaced with fresh medium supplemented with different concentrations of the extracts and test compounds (6.25–400 μg/mL in PBS). Potassium dichromate (K_2_Cr_2_O_7_) was used as a positive control and PBS as a negative control. Cells were incubated with each test material in triplicate for 24 h. The medium was then removed, and the cells were washed twice with PBS and stained with 0.5% crystal violet for 15 min at room temperature. The stain was removed, and the cells were washed in tap water before air-drying at room temperature for 24 h. The dye was then dissolved in methanol and the absorbance at 590 nm was measured in a microplate reader. The results were expressed as IC_50_ values (μg/mL). The cytotoxic activity of the extracts was categorized as follows: IC_50_ ≤ 20 µg/mL = highly cytotoxic, IC_50_ 21–200 µg/mL = moderately cytotoxic, IC_50_ 201–400 µg/mL = weakly cytotoxic, and IC_50_ > 401 µg/mL = no cytotoxicity.

#### 4.7.3. Caco-2 Cell Cytotoxicity Assay

Cytotoxic activity against Caco-2 cells were evaluated using the PrestoBlue cell viability assay (Thermo Fisher Scientific) as previously described [47]. Confluent Caco-2 cells were exposed to different concentrations of extracts or test compounds in culture medium at 37 °C in a 5% CO_2_ atmosphere for 4 or 24 h. Cells were then incubated with 5% (*v*/*v*) PrestoBlue in culture medium as above for 2 h. Fluorescence was measured in an FLx800 fluorescence microplate reader (BioTek Instruments, Winooski, VT, USA) at excitation/emission wavelengths of 560/590 nm. Cell viability was determined relative to an untreated control, after blank correction.

## Figures and Tables

**Table 1:** Industrial chicory and witloof extracts used in the bioactivity assays.

			STL (µg/mg Extract) *			
Code	Raw Material	Method	11β,13-Dihydrolactucin	Lactucin	11β,13-Dihydrolactucopicrin	Lactucopicrin
Wit_H_2_O	Freeze-dried witloof	Water extract (solid–liquid extraction, S-L)	2.13	2.08	1.07	2.23
Wit_EtOAc	Freeze-dried witloof	Ethyl acetate extract (S-L extraction)	0.86	1.35	1.18	3.17
Ci_H_2_O	Fresh chicory	Water extract (S-L extraction)	0.48	1.13	0.39	1.76
Ci_EtOAc	Fresh chicory	Ethyl acetate extract (S-L extraction)	4.81	8.09	3.18	26.88
Ci_SFE	Freeze-dried chicory	Supercritical fluid extraction (SFE)	36.31	109.31	19.50	262.19
Ci_SFE_opt_	Freeze-dried chicory	Optimized SFE	170.19	257.59	55.62	271.04
Ci_SFE_pur_	Freeze-dried chicory	SFE fraction purified by flash column chromatography **	-	-	-	-

* Approximate quantification by using external calibration curves of commercially available standards (n ≥ 2). ** Composed only of a mixture of 8-deoxylactucin and 11β,13-dihydro-8-deoxylactucin.

**Table 2:** Antimicrobial activity of selected pure sesquiterpene lactones and chicory extracts dissolved in DMSO.

	*S. aureus* VTT E-70045	*P. aeruginosa* VTT E- 96728	*E. coli* VTT E-94564^T^
Standards			
Lactucopicrin 4 mM (1.64 mg/mL)	++	+	+
Parthenolide 4 mM (1.00 mg/mL)	++	+	+
11-β-Dihydrolactucopicrin 4 mM (1.65 mg/mL)	++	+	+
Lactucin 4 mM (1.11 mg/mL)	+	+	+
Extracts			
Ci_EtOAc (0.05 mg/mL)	++	+	+
Ci_H_2_O (1.00 mg/mL) *	+	-	NA
Wit_EtOAc (1.00 mg/mL)	++	-	NA
Wit_H_2_O (1.00 mg/mL)	-	-	NA
Ci_SFE (1.00 mg/mL)	+++	-	NA

- no activity, + weak, ++ moderate (0.5 log reduction CFU/mL after 48 h), +++ strong (1.0 log reduction CFU/mL after 48 h); NA, not applicable; * dissolved in ethanol.

**Table 3:** Antibacterial activity of Ci_EtOAc and Ci_H_2_O and pure standard compounds (mg/mL). MIC, minimal inhibitory concentration; MBC, minimal bactericidal concentration.

	*S. aureus* oral		*S. aureus* ATCC 11632		*P. aeruginosa* ATCC 27853		*P. aeruginosa* IBRS P001		*E. coli* ATCC 25922	
Extracts	MIC	MBC	MIC	MBC	MIC	MBC	MIC	MBC	MIC	MBC
Ci_EtOAc	0.75	1.50	0.75	1.50	1.50	3.00	-	-	6.00	6.00
Ci_H_2_O	-	-	-	-	-	-	-	-	-	-
Standards										
Costunolide	-	-	-	-	-	-	0.50	1.00	-	-
Parthenolide	0.08	0.16	0.16	0.31	0.31	0.63	-	-	-	-
Lactucin	-	-	-	-	-	-	-	-	-	-
Lactucopicrin	-	-	0.16	0.31	0.31	0.63	0.50	1.00	-	-
11β, 13-Dihydrolactucin	-	-	-	-	-	-	0.50	1.00	-	-
Controls										
Ampicillin	0.002	0.003	0.002	0.003	0.0002	0.0004	-	-	0.003	0.003
Streptomycin	0.006	0.012	0.050	0.100	0.0004	0.0008	0.050	0.100	0.030	0.030

- No antimicrobial activity with the tested concentrations.

**Table 4:** Antibacterial activities of Ci_SFE_pur_ and Ci_SFE_opt_ (mg/mL). MIC, minimal inhibitory concentration; MBC, minimal bactericidal concentration.

Strain	Ci_SFE_pur_		Ci_SFE_opt_		Ampicillin	
	MIC	MBC	MIC	MBC	MIC	MBC
*Proteus mirabilis* ATCC 7002	0.25	0.50	2.10	4.40	0.01	0.01
*Listeria monocytogenes* NCTC 7973	0.25	0.50	1.05	2.1	0.40	0.50
*Pseudomonas aeruginosa* IBRS P001	0.06	0.12	0.50	1.00	-	-
*Enterobacter cloacae* human isolate	0.25	0.50	2.10	4.40	0.10	0.15
*Yersinia enterocolitica* ATCC 23715	0.25	0.50	2.10	4.40	0.004	0.008
*Klebsiella pneumoniae* ATCC 13883	-	-	0.50	1.00	0.20	0.40
*Campylobacter jejuni* ATCC 33560	0.25	0.50	1.00	2.10	0.02	0.04

- No antimicrobial activity at the tested concentrations.

**Table 5:** Antifungal activity of terpene extracts and pure compounds against (**A**) selected *Candida* species/strains and (**B**) selected other fungal pathogens. MIC, minimal inhibitory concentration; MFC, minimal fungicidal concentration (mg/mL). NA, not applicable.

(A)														
	*C. albicans* 475/15		*C. albicans* ATCC 10231		*C. krusei*		*C. auris*		*C. parapsilosis*					
	MIC	MFC	MIC	MFC	MIC	MFC	MIC	MFC	MIC	MFC				
**Extracts**														
Ci_EtOAc	1.00	2.00	-	-	-	-	-	-	-	-				
Ci_H_2_O	1.00	2.00	-	-	-	-	-	-	-	-				
Ci_SFE_pur_	0.06	0.12	0.06	0.12	0.06	0.12	0.03	0.06	0.25	0.50				
Ci_SFE_opt_	0.50	1.10	0.50	1.10	0.50	1.10	0.25	0.50	0.50	1.10				
**Standards**														
Costunolide	0.50	1.00	-	-	0.13	0.25	-	-	-	-				
Parthenolide	0.25	0.50	-	-	0.03	0.06	-	-	-	-				
Lactucin	1.00	>1.00	-	-	0.50	1.00	-	-	-	-				
Lactucopicrin	1.00	>1.00	-	-	0.50	1.00	-	-	-	-				
11β, 13-Dihydrolactucin	0.50	1.00	-	-	0.50	1.00	0.25	0.50	0.25	0.50				
**Control**														
Ketoconazole	0.030	0.060	0.002	0.032	0.006	0.010	-	-	0.003	0.006				
**(B)**														
	***A. fumigatus***		***A. versicolor***		***A. ochraceus***		***A. niger***		***T. viride***		***P. ochrochloron***		***P. funiculosum***	
	**MIC**	**MFC**	**MIC**	**MFC**	**MIC**	**MFC**	**MIC**	**MFC**	**MIC**	**MFC**	**MIC**	**MFC**	**MIC**	**MFC**
**Extracts**														
Ci_EtOAc	-	-	3.00	4.00	4.00	8.00	6.00	8.00	1.00	2.00	0.50	1.00	NA	NA
Ci_H_2_O	-	-	-	-	-	-	-	-	-	-	-	-	NA	NA
Ci_SFE_pur_	0.13	0.26	0.03	0.06	0.25	0.50	0.50	1.00	NA	NA	NA	NA	0.25	0.50
Ci_SFE_opt_	1.10	2.20	0.50	1.10	1.10	2.20	2.20	4.40	NA	NA	NA	NA	1.10	2.20
**Standards**														
Costunolide	-	-	-	-	-	-	-	-	-	-	-	-	-	-
Parthenolide	-	-	-	-	-	-	-	-	-	-	-	-	-	-
Lactucin	-	-	-	-	-	-	-	-	-	-	-	-	-	-
Lactucopicrin	-	-	-	-	-	-	-	-	-	-	-	-	-	-
11β, 13-Dihydrolactucin	-	-	-	-	-	-	-	-	-	-	-	-	-	-
**Control**														
Ketoconazole	0.180	0.250	0.045	0.062	0.250	0.500	0.375	0.500	0.375	0.500	0.031	0.159	0.150	0.200

- No antifungal activity with the tested concentrations.

**Table 6:** Percentage inhibition of pyocyanin production in *P. aeruginosa* IBRS P001. The standard compounds were applied at 0.5 MIC. The concentrations of pyocyanin in the treated cultures (mg/mL) were compared to an untreated control to determine the percentage inhibition.

Standards	Inhibition (%)	Concentration mg/mL
Costunolide	55.25	0.0054
Lactucin	69.63	0.0036
Lactucopicrin	52.83	0.0057
11β-13-dihydrolactucin	36.13	0.0076
Control	0	0.0120

**Table 7:** Cytotoxicity assessment with *Aliivibrio fischeri*.

Sample Code	EC_50_, mg/L ^a^		Toxicity Category (According to Directive 93/67/EEC) ^b^
	15 min	30 min	
Ci_EtOAc, in ethanol	1133	739	not harmful
Ci_EtOAc, in water	1891	1415	not harmful
Wit_EtOAc	203	184	not harmful
Wit_H_2_O	5737	6385	not harmful
Ci_SFE	83	68	harmful
Ci_SFE_opt_	131	142	not harmful
Ci_SFE_pur_	3076 ^c^	1674 ^c^	not harmful

^a^ Average of two assays. Estimated based on the original concentration in stock. ^b^ According to Directive 93/67/EEC and based on toxicity toward aquatic organisms, compounds can be assigned to the following categories: EC_50_ ≤ 1 mg/L (very toxic); 1 mg/L < EC_50_ ≤ 10 mg/L (toxic); 10 mg/L < EC_50_ ≤ 100 mg/L (harmful); EC_50_ > 100 mg/L (not harmful). ^c^ EC_20_.

**Table 8:** Cytotoxicity of Ci_SFE_opt_ and Ci_SFE_pur_ toward HaCaT cells, presented as the IC_50_ value (µg/mL). K_2_Cr_2_O_7_ was used as a positive control. Results are expressed as mean (±stdev) of triplicates.

	IC_50_ (µg/mL)
Ci_SFE_opt_	303.4 ± 1.5
Ci_SFE_pur_	16.8 ± 0.1
K_2_Cr_2_O_7_	0.9 ± 0.0

## Data Availability

Data is contained within the article and supplementary material.

## Supplementary Materials

The following are available online at www.mdpi.com/article/10.3390/ph14090941/s1, Figure S1: Chromatograms of the commercially available sesquiterpene lactones standards present in *Cichorium intybus* L. analyzed by HPLC-UV at 280 nm: 11β,13-dihydrolactucin (RT = 23.7 min); Lactucin (RT = 27.3 min); 11β,13-dihydrolactucopicrin (RT = 48.6 min) and lactucopicrin (RT = 49.1 min). Figure S2: Antimicrobial activity of 1 mg/mL Wit_EtOAc, Wit_H_2_O and Ci_SFE against *Staphylococcus aureus* VTT E-70045 in liquid cultures incubated for 48 h. Black diamond, microbial control; gray triangle, Wit_EtOAc; black cross, Wit_H_2_O; gray square, Ci_SFE; gray circle, chloramphenicol 50 µg/mL.
