# Supplementary material for: Chicory Extracts and Sesquiterpene Lactones Show Potent Activity against Bacterial and Fungal Pathogens
Source: Pharmaceuticals (Basel). 2021 Sep 20;14(9):941. doi: 10.3390/ph14090941 (PMC8469098; doi:10.3390/ph14090941)
Supplement: Supplementary file 1 [file pharmaceuticals-14-00941-s001.zip › pharmaceuticals-1374423 Supplementary Figure S1.pdf]

A

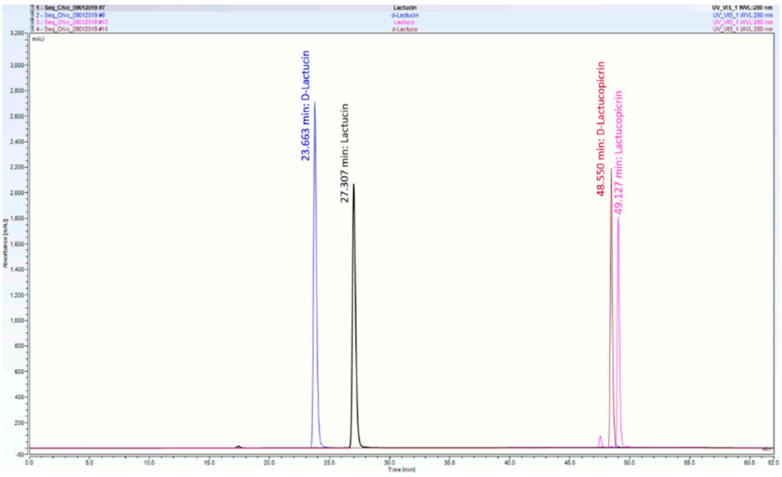

B

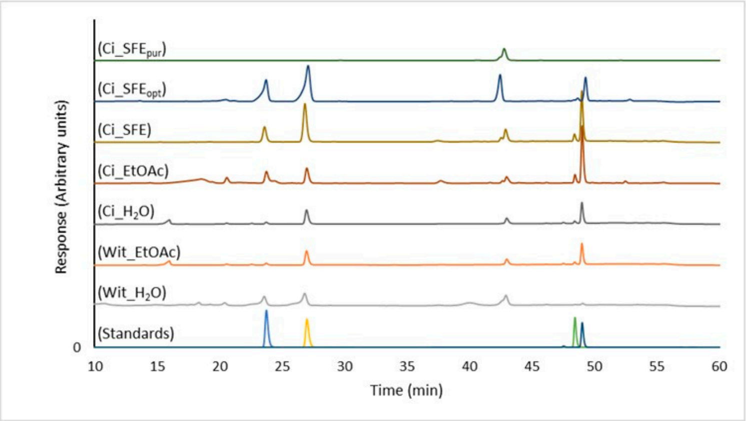

C

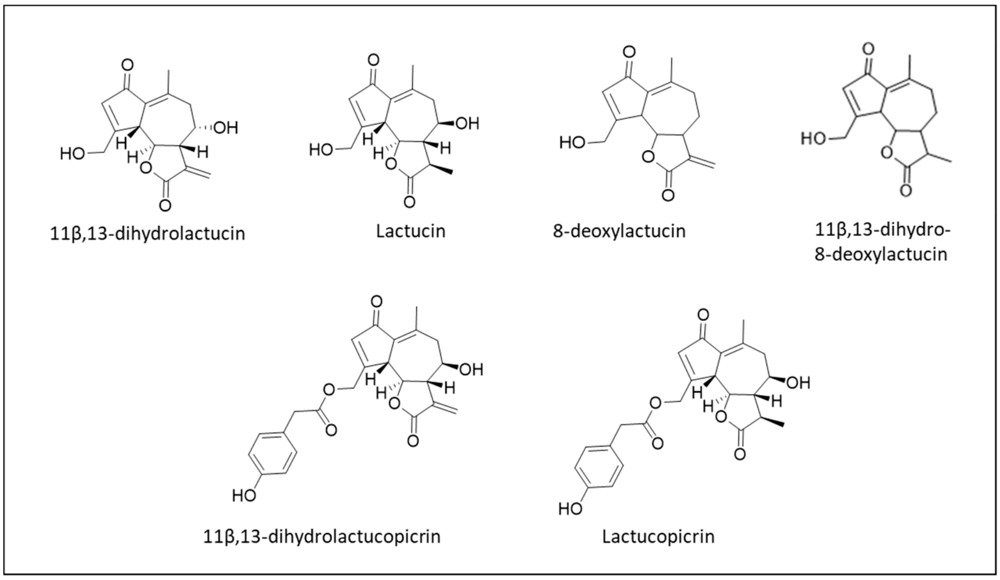

Supplementary Figure S1. A) Chromatograms of the commercially available standards of sesquiterpene lactones, present in *Cichorium intybus* L., analysed by HPLC-UV at 280 nm: 11β,13-dihydrolactucin (Rt: 23.7 min); Lactucin (Rt: 27.3 min); 11β,13-dihydrolactucopicrin (Rt: 48.6 min) and lactucopicrin (Rt: 49.1 min). **B**) HPLC-UV chromatograms of witloof and chicory extracts showing signals at 280 nm. Major sesquiterpenes in the extracts were detected at the following retention times (RT): 11β,13-dihydrolactucin (RT = 23.7 min); lactucin (RT = 27.3 min); 8-deoxylactucin and 11β,13-dihydro-8-deoxylactucin (RT = 43 min), 11β,13-dihydrolactucopicrin (RT = 48.6 min) and lactucopicrin (RT = 49.1 min). Note that no commercial standards are available for 8-deoxylactucin and 11β,13-dihydro-8-deoxylactucin, and they were identified by mass

spectra as previously described by Baixinho et al. (2021). C) Chemical structures of the compounds found in the extracts.
