# Supplementary material for: Chicory Extracts and Sesquiterpene Lactones Show Potent Activity against Bacterial and Fungal Pathogens
Source: Pharmaceuticals (Basel). 2021 Sep 20;14(9):941. doi: 10.3390/ph14090941 (PMC8469098; doi:10.3390/ph14090941)
Supplement: Supplementary file 1 [file pharmaceuticals-14-00941-s001.zip › pharmaceuticals-1374423 Supplementary Figure S2.pdf]

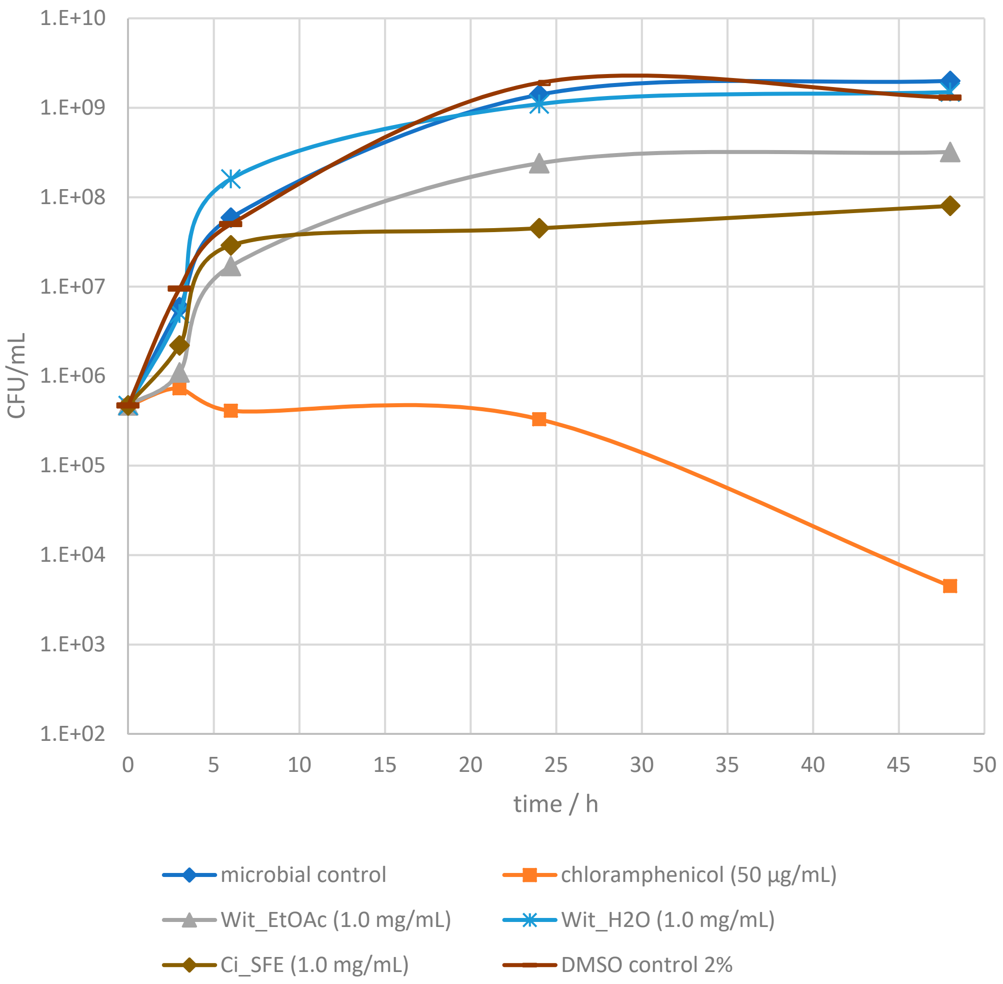

Supplementary Figure S2. Antimicrobial activity of Wit_EtOAc, Wit_H_2O and Ci_SFE against *Staphylococcus aureus* VTT E-70045 at 1 mg/mL concentration in liquid cultures during 48 h of incubation.
